# Genotypes of *Mycobacterium tuberculosis* isolates circulating in Shaanxi Province, China

**DOI:** 10.1371/journal.pone.0242971

**Published:** 2020-12-03

**Authors:** Yan Li, Yu Pang, Tianhua Zhang, Xiaoping Xian, Jian Yang, Rui Wang, Panting Wang, Meng Zhang, Wei Chen

**Affiliations:** 1 Clinical Laboratory, The First Affiliated Hospital, School of Medicine, Xi’an Jiaotong University, Xi’an, China; 2 Clinical Laboratory, Shaanxi Provincial Institute for Tuberculosis Control and Prevention, Xi’an, China; 3 National Clinical Laboratory on Tuberculosis, Beijing Chest Hospital, Capital Medical University, Beijing Tuberculosis and Thoracic Tumor Institute, Beijing, China; University of Minnesota, UNITED STATES

## Abstract

**Objectives:**

The prevalence of drug-resistant TB in Shaanxi Province is higher than other areas. This study was aimed to investigate the genetic diversity and epidemiology of Mycobacterium tuberculosis clinical strains in Shaanxi Province, China.

**Methods:**

From January to December 2016, a total of 298 Mycobacterium tuberculosis clinical isolates from smear-positive pulmonary tuberculosis patients were genotyped by Mcspoligotyping and 15-locus VNTR.

**Results:**

We found that the Beijing family strains was the most prominent family(81.54%, 243/298). Other family strains included T family(9.06%, 27/298), U family(0.67%, 2/298), LAM9 family(0.34%, 1/298) and Manu family(0.34%, 1/298). The rates of multidrug-resistant (MDR) *M*.*Tuberculosis*, age, type of case and education between Beijing and non-Beijing family strains were not statistically different, while the distribution in the three different regions among these was statistically significant.

VNTR results showed that strains were classified into 280 genotypes, and 33 (11.07%) strains could be grouped into 14 clusters. 11 of the 15-VNTR loci were highly or moderately discriminative according to the Hunter-Gaston discriminatory index.

**Conclusions:**

We concluded that the Beijing family genotype was the most prevalent genotype and 15-locus VNTR typing might be suitable for genotyping of *M*. *tuberculosis* in Shaanxi Province. There was less association between Beijing family genotypes and drug resistance in our study area.

## 1. Introduction

Tuberculosis(TB) is a chronic respiratory infectious diseases caused by *Mycobacterium tuberculosis*(*M*. *tuberculosis*), which is one of the most health-threatening diseases in the world. According to the World Health Organization (WHO)’s report, almost two-thirds of these new cases were in India, China, Indonesia, the Philippines, Pakistan, Nigeria, Bangladesh and South Africa [1]. The WHO estimates that there were about 866,000 new cases of TB patients in China, which has decreased compared with last year, but the epidemic situation of tuberculosis still can’t be ignored. Shaanxi is a province located in north-western China, with an area of 210,000 square kilometers and a population of 38.7 million in 2019.The topography of Shaanxi province from north to south is loess Plateau, Guanzhong plain and Qinba mountains. The development of the three regions is uneven, and the economies of northern and southern Shaanxi are relatively backward. The large population density and high mobility of the population in the middle Shaanxi region are prone to the spread and epidemic risk of tuberculosis to a large extent. The total incidence of TB in Shaanxi province is 56 cases per 100,000 population, according to the Provincial TB Surveillance and Reporting System. According to the data from drug resistance TB baseline survey in Shaanxi Province [2], the rate of drug resistance TB and the multi-drug resistance TB were 30.34% and 5.94% respectively. It is reported in the literature that MDR accounts for 3.5% (38/1086) of initial treatment cases and 12.09% (52/430) of retreatment cases [2]. Therefore, Shaanxi Province is experiencing a severe challenge in the TB epidemic.

Rapid and useful genotyping is a method of molecular epidemiology based on the analysis of characteristics of nucleic acid [3]. It has been proved that molecular epidemiology to be a useful tool for the epidemic, transmission, gene polymorphism and genetic relationship of *M*. *tuberculosis* [3]. Genotyping methods can detect multiple *M*. *tuberculosis* clinical samples from different sources, understand the dynamic of disease transmission, and control the outbreak of tuberculosis effectively [4], such as variable number of tandem repeat (VNTR), spacer oligonucleotide typing (Spoligotyping) and whole genome sequencing facilitates(WGS) at present [5–7]. Mcspoligotyping based on multicolor melting curve analysis is different from spoligotyping [8]. In this study, we used Mcspoligotyping and VNTR to type a collection of *M*. *tuberculosis* isolates of our area. Because of the molecular epidemiology technologies, lots of studies shown that the Beijing family strains of *M*. *tuberculosis* were highly prevalent strains in China, Japan, southeast Asia, Russia and other countries and regions [9,10]. And some studies have demonstrated that genotype diversity of *M*. *tuberculosis* was associated with drug resistance [11–14]. However, the proportion of Beijing genotypes in different regions is quite different. It is necessary to study genotypic diversity and epidemiology of *M*. *tuberculosis* clinical isolates in different regions.

Shaanxi's drug-resistant epidemic situation remains severe, and it faces many challenges such as multidrug resistance and rifampicin resistance. However, there are few reports on the molecular epidemiology of *M*. *tuberculosis* in Shaanxi Province. In our study, we combined spoligotyping and VNTR to analyse the genotypes of *M*. *tuberculosis* strains collected in Shaanxi during 2016 and to find the relationships between genotype and drug-resistance.

## Materials and methods

### 2.1 Ethical statement

The study was conducted in accordance with the principles of the Declaration of Helsinki, and the study protocol was approved by the ethics review board of Shaanxi Provincial Institute for Tuberculosis Control and Prevention. We have obtained written informed consent from all study participants. Minors (age <18 years old) have obtained the consent of their parents or guardians.

### 2.2 Bacterial isolates

All the smear-positive sputum samples obtained from pulmonary TB patients assigned in different prefectures were continuously collected from Jan 1 to Dec 30, 2016, including Xi’an, Xianyang, Baoji, Weinan, Tongchuan, Hanzhong, Ankang, Shangluo, Yulin and Yan’an. Consecutive specimens were obtained from patients who were diagnosed with TB according to the national guide lines of China. Authors had access to information that could identify individual participants during or after data collection. Samples from each patient were cultivated on Löwenstein-Jensen (L-J) medium following WHO guidelines [15]. The standard strain of *M*. *tuberculosis* (H37Rv) was donated by the National TB Reference Laboratory of China.

### 2.3 Species identification and drug susceptibility testing

All isolates were identified by fluorescent PCR melting curve method using the MeltPro TB assay (Zeesan Biotecheh, China) according to manufacturer’s protocol [8]. The proportional method was used for determination of in vitro drug susceptibility of *M*. *tuberculosis* as previously described [16]. The concentration of isoniazid (INH), rifampicin(RIF), ethambutol (EMB), streptomycin (SM), ofloxacin (OFLX), kanamycin (KAN) in the solid medium were 0.2μg/ml, 40μg/ml, 2μg/ml, 4μg/ml, 4μg/ml, 30μg/ml, respectively.

### 2.4 DNA extraction

Using magnetic beads method, genomic DNA was performed with an automated DNA extraction instrument (Zeesan Biotecheh, China) according to the manufacturer’s protocol. After the program is completed, the supernatant is collected as the template DNA for subsequent genotyping and stored at−20°C for further analysis.

### 2.5 Mcspoligotyping

Spoligotyping was performed using commercial Mcspoligotyping kit manufactured by MeltPro TB assay (Zeesan Biotecheh, China) according to the manufacturer’s protocol. The technology based on multicolor melting curve analysis (MMCA), which is new detection method based on real-time polymerase chain reaction (PCR) [17]. The 43 spacers were detected by 43 fluorescent probes that could be specifically hybridized with them, with corresponding fluorescent and melting temperature (Tm) respectively. By the end of the experiment, the analysis software can automatically export the fluorescence and Tm of 43 intervals of each sample. The results were given a corresponding 43-digit binary code in the order of Sp1 to Sp43. The results of genotyping are indicated by 43-digit binary code, where 1 indicates the presence of the spacer and 0 indicates its absence. The original binary data submitted to the SpolDB 4.0 database (http://www.pasteur-guadeloupe.fr:8081/SITVIT_ONLINE/query), in order to compared to the SITVIT database to obtain the spoligotype international types(SITs) and clades of *M*. *tuberculosis* isolates [18].

### 2.6 VNTR

Strains of *M*. *tuberculosis* were genotyped by 15 VNTR loci (Mtub04, Mtub21, Mtub30, Mtub39, Qub-11b, Qub-26, Qub-4156, MIRU4, MIRU40, MIRU10, MIRU16, MIRU26, MIRU31, ETRA, ETRC) as previously reported [19]. The amplified products were analyzed by 2% agarose electrophoresis. To determine the sizes of the amplicons with software (Quantity One 4.6.2). The 100 bp DNA ladder and amplicons of H37Rv were as a marker to calculate the repeat number of each locus. The results of VNTR genotyping were obtained using http://www.miru-vntrplus.org/MIRU/index.faces. Using the Hunter-Gaston discriminatory index (HGI) to evaluate the allelic diversity of the different VNTR loci. In addition, the discriminatory powers were divided into high (HGI>0.6), moderate (0.3≤HGI≤0.6) or low (HGI<0.3), according to the score of each VNTR locus [20].

$$HGI=1-\frac{1}{N(N-1)}\sum_{j=1}^{s}n_{j}\left(n_{j}-1\right)$$

### 2.7 Data analysis

The genotyping in binary format were entered in an Excel spreadsheet and clustered with BioNumerics software version 5.0 (Applied Maths, Sint-Martens-Latem, Belgium). All statistical analyses were performed using the SPSS software (version 18.0). Chi-square test was used to detect significant differences between the two groups. P<0.05 was considered significant.

## Results

### 3.1 Study population

A total of 300 clinical culture-positive isolates were enrolled in this study. Of these 300 strains, 298 strains were identified as *M*. *tuberculosis*, while the other 2 strains were non tuberculous mycobacteria (NTM). Overall, there were 213 male and 85 female patients with an average age of 41.5years old. As a consequence, 298 *M*. *tuberculosis* isolates were further analysed by molecular epidemiological methods.

### 3.2 Mcspoligotyping

In order to investigate the genotype diversity of prevalent strains in Shaanxi province, we used Mcspoligotyping method to analyze 298 strains from different districts. In these strains, the Beijing genotype families were 243 strains, accounting for 81.54% (243/298) and non-beijing genotype families were 55 strains, accounting for 18.46% (55/298). Among the Beijing family strains, 224 strains (75.17%) were typical Beijing family strains, and 19 (6.38%) were atypical Beijing family strains. The non-Beijing family strains included 27 from T family(9.06%), 2 from U family(0.67%), 1 from LAM9 family(0.34%), 1 from Manu family(0.34%), 1 from undefined (0.34%) and 23 orphans type which is not found in SITVITWEB database. In addition, we submitted the data to the MIRU-VNTR plus web database for cluster analysis (http://www.miru-vntrplus.org/MIRU/index.faces). The 298 strains can be divided into 13 clusters, with a clustering rate of 85.91% (256/298). The remaining 29 strains were not been clustered, accounting for 9.73% (29/298) (S1 Table).

### 3.3 Demographic and regional distributive characteristics of Beijing and Non-Beijing *M*. *tuberculosis* isolates

Demographic information of 298 patients including age, sex, previous TB history, educational background are summarized in Table 1. In order to analyzed relationship between the Beijing family genotypes and sex, age, case type, educational level, we compared regional distributive and demographic Characteristics of Beijing and Non-Beijing *M*. *tuberculosis* isolates in this study. We found that the proportion of male infections with Beijing family genotype strains was significantly higher than that of female infections(χ^2^ = 6.47>χ^2^_0.05,1_ = 3.84, P<0.05). The percentage of Middle Shaanxi region patients infected with Beijing genotype strains was significantly higher than that of Southern and Northern Shaanxi patients(χ^2^ = 6.53>χ^2^_0.05,2_ = 5.99, P<0.05). Compared with the distribution of Beijing family isolates from patients in Southern Shaanxi region, those from Middle Shaanxi region patients [OR (95% CI): 0.419 (0.213~0.825), P = 0.012] were significantly lower (Table 1).

**Table 1:** Characteristics of Beijing and non-Beijing genotype *M*. *tuberculosis* isolates enrolled in this study.

Characteristics	Number (%) of strains			*OR*	*95%CI*	*P*
	Total n = 298	Beijing n = 243	Non-Beijing n = 55			
**Gender**						0.014
**Male**	213(71.48)	166(68.31)	47(85.45)	1		
**Female**	85(28.52)	77(31.69)	8(14.55)	0.367	0.165~0.814	
**Age group**						0.176
**<30**	107(35.91)	93(38.27)	14(25.45)	1		
**30~60**	137(45.97)	109(44.86)	28(50.91)	1.706	0.849~3.432	0.134
**>60**	54(18.12)	41(16.87)	13(23.64)	2.106	0.910~4.877	0.082
**Type of case**						0.533
**New case**	267(89.60)	219(90.12)	48(87.27)	1		
**Re-treated**	31(10.40)	24(9.88)	7(12.73)	1.331	0.542~3.267	
**Level of education**						0.467
**Illiteracy and semiliterate**	17(5.70)	13(5.35)	4(7.27)	1.179	0.325~4.277	0.802
**Primary school**	58(19.46)	46(18.93)	12(21.82)	1		
**Junior high school**	69(23.15)	53(21.81)	16(29.09)	1.157	0.496~2.697	0.735
**High school and technical secondary school**	121(40.60)	101(41.56)	20(36.36)	0.759	0.342~1.683	0.497
**College and university or above**	33(11.07)	30(12.35)	3(5.45)	0.383	0.100~1.473	0.163
**Region**						0.036
**Southern Shaanxi (Hanzhong, Ankang, Shangluo)**	90(30.20)	67(27.57)	23(41.82)	1		
**Northern Shaanxi (Yulin,Y an’an)**	60(20.13)	47(19.34)	13(23.64)	0.794	0.365~1.725	0.560
**middle Shaanxi (Xi’an, Xianyang, Weinan, Tongchuan, Baoji)**	148(49.66)	129(53.09)	19(34.55)	0.419	0.213~0.825	0.012
**Resistant**						0.185
**Non-resistance**	211	168	43	1		
**Resistant**	87	75	12	0.625	0.312~1.253	

### 3.4 Relationship between Beijing family genotypes and drug resistance

We further analyzed the relationship between spoligotypes and drug resistance among *M*. *tuberculosis* strains enrolled in this study. As shown in Table 2, of 243 isolates belonged to Beijing genotype, the rates of resistant isolates were 18.52% (45/243) for SM, 21.40% (52/243) for INH, 16.46% (40/243) for RFP, 6.17% (15/243) for EMB, 2.88% (7/243) for KM, 10.29% (25/243) for OFX, 13.17% (32/243) for MDR, respectively. For non-Beijing genotype strains, 10.91%(6/55) were INH resistant, 10.91%(6/55) were resistant to RIF, 3.64%(2/55) were resistant to EMB,10.91%(6/55) were SM resistant, 3.64%(2/55) were resistant to Km and 3.64%(2/55) were OFX resistant. Although a higher proportion of INH, RFP, SM, OFX resistance was observed in Beijing genotype strains relative to non-Beijing genotype strains, statistical analysis revealed that there was no statistical difference.

**Table 2:** Genotype distributions of clinical isolates in resistance to antituberculosis drugs.

Genotype	No	Resistance to (No. of isolates, %)						MDR (No, %)	Sensitivity
		SM	INH	RFP	EMB	KM	OFX		
**Beijing**	243	45 (18.52)	52 (21.40)	40 (16.46)	15 (6.17)	7 (2.88)	25 (10.29)	32 (13.17)	168 (69.14)
**Non-Beijing**	55	6 (10.91)	6 (10.91)	6 (10.91)	2 (3.64)	2 (3.64)	2 (3.64)	4 (7.27)	43 (78.18)
**χ**^**2**^		1.83	3.15	1.06	0.17^a^	0.02^a^	1.67^a^	1.47	1.49
***P***		>0.05	>0.05	>0.05	>0.05	>0.05	>0.05	>0.05	>0.05

^a^ Representing corrected chi square test.

### 3.5 VNTR

**Fig 1:**
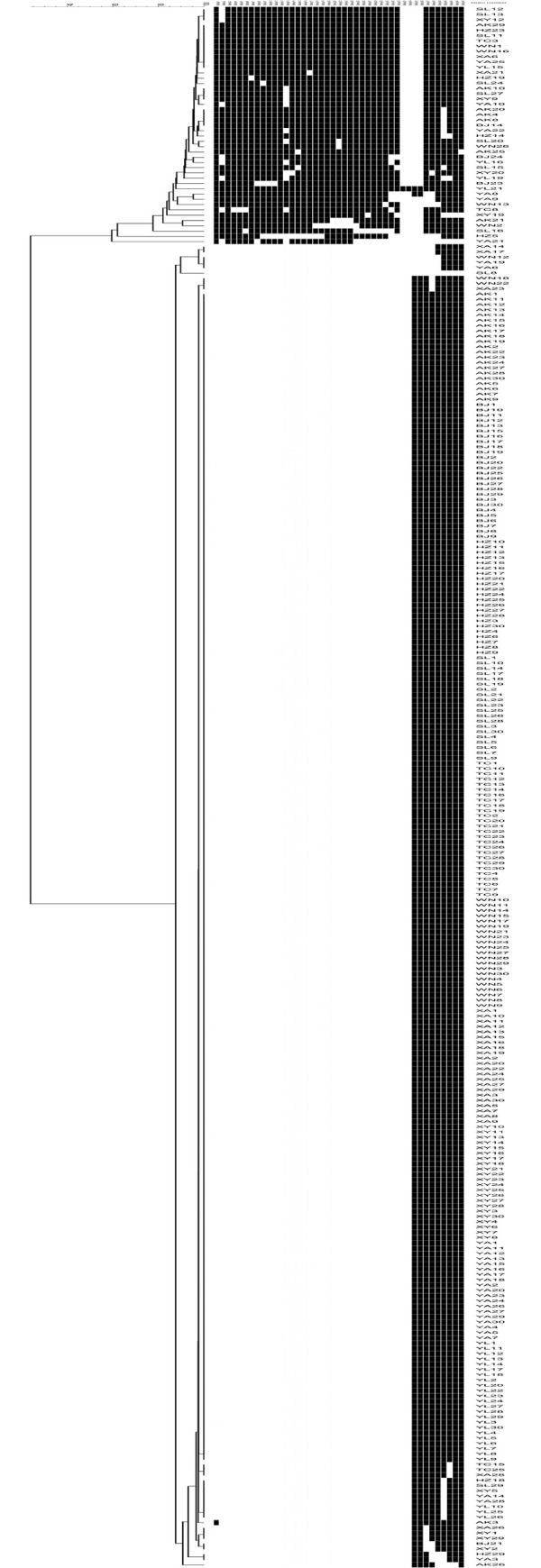
Dendrogram showing the clustering by Mcspoligotyping and 15-Loci VNTR of 298 *M*. *tuberculosis* strains from Shaanxi Province.

The 298 strains were classified into 280 genotypes, and 33 (11.07%) strains could be grouped into 14 clusters, including 2 to 5 isolates per cluster in Fig 1. The remaining 265 strains were unique. As summarized in Table 3, MIRU26、QUB11b、Mtub04、Mtub21 had high discriminating ability, the HGI scores of which were higher than 0.6. Additionally, Qub26, MIRU10, Mtub39, MIRU31, Qub4156, ETRA, MIRU40 showed moderate discrimination (0.3≤HGDI≤0.6). The remaining 4 VNTR loci, including Mtub30、ETRC、MIRU4、MIRU16 sites had low resolution (HGI < 0.3). We also analyzed the discriminating ability of VNTR loci between Beijing and non-Beijing genotypes. There were 12 loci had good discriminatory power in non-Beijing family strains. Compared with non-Beijing genotype strains, only 8 loci in Beijing family strains had good discriminatory discriminatory power (Table 3).

**Table 3:** Polymorphism analysis of 15 VNTR loci in 300 strains of *M*. *tuberculosis*.

VNTR locus	VNTR alias	HGI		
		All strains	Beijing	Non-Beijing
**2163b**	QUB11b	0.741	0.694	0.763
**1955**	Mtub21	0.672	0.602	0.691
**4052**	QUB26	0.546	0.534	0.583
**4156**	QUB4156	0.348	0.350	0.329
**2996**	MIRU26	0.814	0.806	0.759
**424**	Mtub04	0.697	0.698	0.682
**2165**	ETRA	0.344	0.223	0.595
**802**	MIRU40	0.332	0.255	0.567
**3690**	Mtub39	0.422	0.366	0.575
**3192**	MIRU31	0.426	0.267	0.635
**960**	MIRU10	0.459	0.327	0.461
**580**	MIRU04	0.119	0.068	0.285
**577**	ETRC	0.125	0.114	0.156
**1644**	MIRU16	0.112	0.076	0.243
**2401**	Mtub30	0.278	0.083	0.402

## Discussion

To date, several molecular genetic methods are available, including IS*6110* restriction fragment length polymorphism(IS6110-RFLP), spoligotyping, MIRU-VNTR and WGS. IS6110 RFLP used to be the gold standard for M. tuberculosis genotyping but it is time consuming, labor intensive and provides limited resolution for low IS6110 copy numbers, makes it less useful for long-term prospective studies [21]. WGS is the latest typing technology, however, due to the high demand for technical personnel, restricting its wide use in resource-limited countries. Spoligotyping and MIRU-VNTR are the traditional typing methods in molecular epidemiological studies of *M*. *tuberculosis*. The traditional spoligotyping process involves amplification, hybridization, incubation, signal development and other steps which based on a homemade membrane. The operation is complicated and the whole procedure takes at least 8 hours. Mcspoligotyping, is an innovative molecular test for genotyping of *M*. *tuberculosis*, which developed by Zeesan Biotecheh (Xiamen, China) [8].Unlike previous spoligotyping technology, this method is based on melting curve analysis with dually labeled probes to process the detection of 43 interval sequences, and then complete the genotyping of *M*. *tuberculosis*. Traditional operation of membrane hybridization is complex and easy to cause carry over pollution. Compared with traditional spoligotyping, McSpoligotyping results can be operated within 3h by a single step of DNA addition [8].

The Beijing family of *M*. *tuberculosis* strains are the most prevalent strains in China, as well as worldwide. As described in previously reports, Beijing family accounts for 54.50% to 92.59% of the *M*. *tuberculosis* strains [22]. While, this was the first report on the molecular epidemiological of *M*. *tuberculosis* strains in Shaanxi Province. Our results showed that the Beijing family strains accounted for 81.54% (243/298) of all the strains circulating in Shaanxi, which was similar to the findings from northern China, such as Henan (80.00%) [22], Jiangsu(80.4%) [23], Heilongjiang(89.5%) [24], Beijing(82.0%) [25]. But it was higher than those from south China, such as Yunnan (55.7%) [26], Guangdong(25.0%) [27], Fujian(54.5%) [28]. In consistent to our results, a previous molecular epidemiological study on the basis of national survey revealed that the proportion of Beijing family genotype in the northern area is higher than that in the southern area.

In line with previous observation, the most prevalent in 298 strains of genotype was SIT1 (Beijing family), followed by SIT53 (T1 family) and SIT190 (Beijing family). We also observed that the minority strains belong to non-Beijing families, including T1, T2, T3, Manu_ancestor, LAM9 and U families. According to literature reports [29], It is well described that he T family genotype is predominantly prevalent in Africa, Central, South America and Europe, and we found this genotype in our research. While The LAM family was also found in in Jiangsu [23], Yunnan province [26], Xinjiang autonomous region [30], and Taiwan [31] in China. As this is the first report of the predominant genotype of *M*. *tuberculosis* strains in Shaanxi province, we need more research in this field.

This study showed that the most prevalent in 298 strains of genotype was SIT1 (Beijing family), followed by SIT53 (T1 family) and SIT190 (Beijing family), which was consistent to the report in most areas of China [22].

Due to the high prevalence of Beijing genotypes in many areas, some researchers believe Beijing genotypes may be associated with high virulence and spread [13]. In our study, we found that there were significant differences in gender and geographical distribution among the Beijing genotype strains.Some published studies have revealed that Beijing genotype was related to drug resistance [13,32,33], however, opposite data were declared by other researchers that there is no association between them [24,34,35]. We found that the resistance rates of SM, INH, RFP, EMB, KM, OFX and MDR of Beijing family genotypes were all higher than those of non-Beijing, while no significant different was observed due to small sample size. Further study is urgently needed to elucidate the relationship between Beijing genotype strains and drug resistance by the recruitment a large number of *M*. *tuberculosis* strains in Shaanxi Province. This study also analyzed the resistance of ofloxacin and kanamycin anti-tuberculosis drugs at that time. In the future research process, we will keep consistent with the latest anti-tuberculosis drugs recommended by WHO. In our study, the statistical analysis showed that there was no significant difference between the Beijing and non-Beijing genotype strains in non-resistance and resistance groups, indicating that the Beijing genotype was less likely to be associated with drug resistance in our area.

It has been proved that Mcspoligotyping was more accurate than spoligotyping to distinguish Beijing genotypes from non Beijing genotypes [8], but spoligotyping typing method had a poor discriminability to distinguish among Beijing genotype strains. Therefore, we further applied the most commonly used 15 loci VNTR to this study [19,36]. The Mcspoligotyping grouped 269 strains into 13 clusters and the cluster rate was 90.27%. While, VNTR typing in our study clustered 14 of 33 strains, the cluster rate of the which was 37.30%. Moreover, 243 strains of Beijing family strain can be divided into 231 genotypes using the VNTR typing method, nearly each strain of Beijing genotype can become a clade. The results showed that the alleles of different VNTR loci showed great differences in each strains. Spoligotyping method can be used as a first-line typing technique.For non-beijing family strains, there are 12 loci with good or moderate discriminatory power, while in the Beijing family strain, only 8 loci are good or moderate. In this study, we think the 15-site VNTR typing method is suitable for genotyping of strains in Shaanxi Province, but not suitable for all Beijing genotypes. It is suggested that in future studies, we should remove the low discriminatory power of loci and continue to discover more appropriate loci to improve the resolution. Thus, the results of this study may provide a reference for future selection of VNTR loci for *M*.*tuberculosis* in our province.

In conclusion, our data reveal that Beijing family strains are predominantly prevalent in Shaanxi province and the majority of the epidemic strain belongs to the SIT1 genotype. In addition, the 15-loci VNTR typing method is suitable for genotyping of strains in this area. Further study is urgently needed to elucidate the transmission of Beijing genotype and the relationship between Beijing genotype strains and drug resistance by the recruitment a large number of M. *tuberculosis* strains in Shaanxi Province. Next, our study will focus on the whole genome sequencing technology to carry out the genotyping of *M*. *tuberculosis* in our region.

## Supporting information

S1 Table Spoligotypes of 298 *M*. *tuberculosis* collected from Shaanxi in this study. (DOC)
