# Supplementary material for: Genotypes of Mycobacterium tuberculosis isolates circulating in Shaanxi Province, China
Source: PLoS One. 2020 Dec 3;15(12):e0242971. doi: 10.1371/journal.pone.0242971 (PMC7714122; doi:10.1371/journal.pone.0242971)
Supplement: S1 Table — (DOC) [file pone.0242971.s001.doc]

**S1 Table. Spoligotypes of 298 *M. tuberculosis* collected from Shaanxi in this study**

| **Family^a^** | **SIT^b^** | **spoligotype^c^** | **Number(%)** |
| --- | --- | --- | --- |
| **Beijing** | 1 | 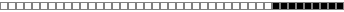 | 224(75.17) |
| **Beijing** | 190 | 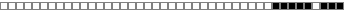 | 8(2.68) |
| **Beijing** | 632 | 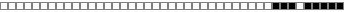 | 3(1.01) |
| **Beijing** | 265 | 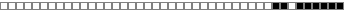 | 3(1.01) |
| **Beijing** | 941 | 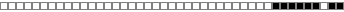 | 3(1.01) |
| **Beijing** | 260 | 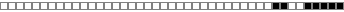 | 2(0.67) |
| **T1** | 53 | 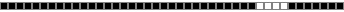 | 9(3.02) |
| **T1** | 334 | 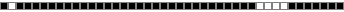 | 3(1.01) |
| **T1** | 281 | 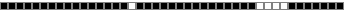 | 1(0.34) |
| **T1** | 926 | 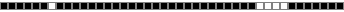 | 1(0.34) |
| **T1** | 86 | 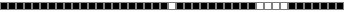 | 1(0.34) |
| **T1** | 78 | 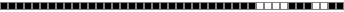 | 1(0.34) |
| **T1** | 1129 | 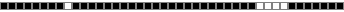 | 1(0.34) |
| **T1** | 717 | 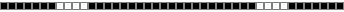 | 1(0.34) |
| **T2** | 52 | 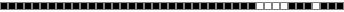 | 4(1.34) |
| **T3** | 37 | 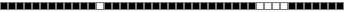 | 3(1.01) |
| **T3** | 504 | 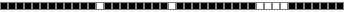 | 1(0.34) |
| **Ambiguous:T3 T2** | 73 | 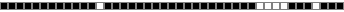 | 1(0.34) |
| **Manu_ancestor** | 523 | 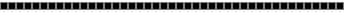 | 1(0.34) |
| **LAM9** | 42 | 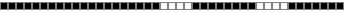 | 1(0.34) |
| **U** | 616 | 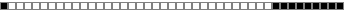 | 1(0.34) |
| **U** | 238 | 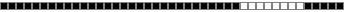 | 1(0.34) |
| **Undefined** | 2669 | 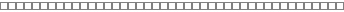 | 1(0.34) |
| **Orphan** | | 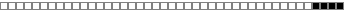 | 3(1.01) |
| **Orphan** | | 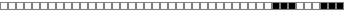 | 2(0.67) |
| **Orphan** | | 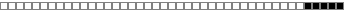 | 2(0.67) |
| **Orphan** | | 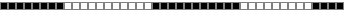 | 1(0.34) |
| **Orphan** | | 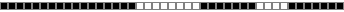 | 1(0.34) |
| **Orphan** | | 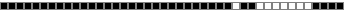 | 1(0.34) |
| **Orphan** | | 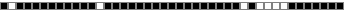 | 1(0.34) |
| **Orphan** | | 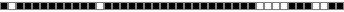 | 1(0.34) |
| **Orphan** | | 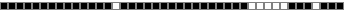 | 1(0.34) |
| **Orphan** | | 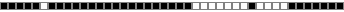 | 1(0.34) |
| **Orphan** | | 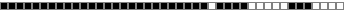 | 1(0.34) |
| **Orphan** | | 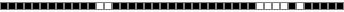 | 1(0.34) |
| **Orphan** | | 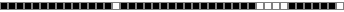 | 1(0.34) |
| **Orphan** | | 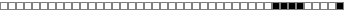 | 1(0.34) |
| **Orphan** | | 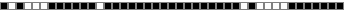 | 1(0.34) |
| **Orphan** | | 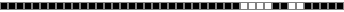 | 1(0.34) |
| **Orphan** | | 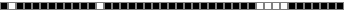 | 1(0.34) |
| **Orphan** | | 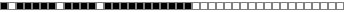 | 1(0.34) |
| **Orphan** | | 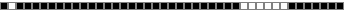 | 1(0.34) |

^a^ Representing spoligotype families as assigned in SITVITWEB database.

^b^ SIT from SITVITWEB database.

^c^ “■” indicates the presence of the spacer, “◻” indicates the absence of the spacer.
